# Novel targets in rectal cancer by considering lncRNA–miRNA–mRNA network in response to *Lactobacillus acidophilus* consumption: a randomized clinical trial

**DOI:** 10.1038/s41598-022-13297-9

**Published:** 2022-06-02

**Authors:** Zohreh Khodaii, Mahboobeh Mehrabani Natanzi, Solmaz Khalighfard, Maziar Ghandian Zanjan, Maryam Gharghi, Vahid Khori, Taghi Amiriani, Monireh Rahimkhani, Ali Mohammad Alizadeh

**Affiliations:** 1 Dietary Supplements and Probiotic Research Center, Alborz University of Medical Sciences, Karaj, Iran; 2 Evidence-Based Phytotherapy and Complementary Medicine Research Center, Alborz University of Medical Sciences, Karaj, Iran; 3 Department of Biology, Science and Research Branch, Islamic Azad University, Tehran, Iran; 4 Division of Gastroenterology Hepatology and Nutrition, Department of Medicine, College of Medicine, University of Florida, Gainesville, FL USA; 5 Ischemic Disorders Research Center, Golestan University of Medical Sciences, Gorgan, Iran; 6 Faculty of Allied Medical Sciences, Tehran University of Medical Sciences, Tehran, Iran; 7 Cancer Research Center, Cancer Institute, Tehran University of Medical Sciences, Tehran, Iran; 8 Breast Disease Research Center, Cancer Institute, Tehran University of Medical Sciences, Tehran, Iran

**Keywords:** Cancer, Cell biology, Computational biology and bioinformatics, Microbiology, Molecular biology, Biomarkers, Diseases, Medical research, Molecular medicine, Oncology

## Abstract

We aimed to explore the lncRNA–miR–mRNA network in response to *Lactobacillus* *acidophilus* (*L. acidophilus*) consumption in rectal cancer patients. The candidate miRs were first taken from the GEO and TCGA databases. We constructed the lncRNA–miR–mRNA network using the high-throughput sequencing data. At last, we created a heatmap based on the experimental data to show the possible correlation of the selected targets. The expression levels of selected targets were measured in the samples of 107 rectal cancer patients undergoing placebo and probiotic consumption and 10 noncancerous subjects using Real-Time PCR. Our analysis revealed a group of differentially expressed 12 miRs and 11 lncRNAs, and 12 genes in rectal cancer patients. A significant expression increase of the selected tumor suppressor miRs, lncRNAs, and genes and a substantial expression decrease of the selected oncomiRs, onco-lncRNAs, and oncogenes were obtained after the probiotic consumption compared to the placebo group. There is a strong correlation between some network components, including miR-133b and IGF1 gene, miR-548ac and MSH2 gene, and miR-21 and SMAD4 gene. In rectal cancer patients, *L.* *acidophilus* consumption was associated with improved expression of the lncRNA–miR–mRNA network, which may provide novel monitoring and therapeutic approaches.

## Introduction

Colorectal cancer (CRC) is the third most common cancer, ranked as the second cause of cancer-related death with 9.4% of the total cancer deaths^1^. Accordingly, there is a complex association between gut microflora, cancer development, and treatment response of CRC^2,3^. Therefore, interventions targeting the gut microbiome can offer a clinical application for cancer prevention and treatment. The cross-talk between the gut microbiome and host is mediated by metabolites, proteins, and non-coding RNAs^4,5^. Recent studies suggested that the gut microbiota can influence the miRs' expression pattern, leading to intestinal homeostasis^4,6^. On the other hand, the microRNAs (miRs) can shape the gut microbiome^5,7^. However, the expression of non-coding RNAs can be modulated by other factors such as diet.

The mechanisms by which some dietary factors modify non-coding RNAs' expression, including miRs and long non-coding RNAs (lncRNA), can lead to the modulation of the gut microbiota and the inhibition of tumor growth^8^. Yuan et al. presented the integrated expression analysis of miRs and intestinal microbiome profiles in CRC patients^9^. Their findings enlightened the highly interconnected network between miRs and microbiome composition and supported the miRs' role in mediating host-microbial interaction in rectal cancer^9^. In the previous study, we demonstrated that the consumption of the probiotics, such as *Lactobacillus* *acidophilus* (*L.* *acidophilus*) and Bifidobacterium *bifidum,* could increase the expression of the tumor suppressor miRs and decrease the oncogenes and their target genes in an animal model of colon cancer^10^. Likewise, Rodríguez-Nogales et al. reported that probiotic consumption could improve the expression of miR-155 and miR-223 in an animal model of colitis^11^. Similarly, Gianotti et al. showed that the low dose administration of *L.* *acidophilus* could improve health status and the immune system function in CRC patients^12^.

It has been comprehended that lncRNAs could interact with miRs and might regulate their target gene expression. This phenomenon has been known as the sponge-like effect of lncRNAs and is explained in the competitive endogenous RNAs (ceRNA) hypothesis. The ceRNA networks have revealed a new mechanism of interactions between RNAs and play fundamental roles in several biological processes and the progress of neoplasms. They might serve as diagnostic and prognosis biomarkers and even therapeutic targets.

In this setting, lncRNAs can bind to protein-encoding gene sequences to form triple RNA–DNA complexes, suppress gene expression, interact with proteins, and develop nucleic acid–protein interactions^9^. A complex correlation between coding and non-coding RNAs has been observed in different malignancies, including CRC^13,14^. Therefore, it is vital to investigate the interactions and mechanisms in regulatory networks, including lncRNAs, miRs, mRNAs, genetic mutations, and epigenetic modifications in rectal cancer. We believe that the new biomarkers will be revealed to diagnose and develop new treatment modalities for rectal cancer. Consequently, we aimed to investigate the profile of the lncRNA–miR–mRNA network in response to *L.* *acidophilic* consumption in patients with non-metastatic rectal cancer.

## Results

### Identification of differentially expressed miRs, lncRNAs, and mRNAs

**Figure 1:**
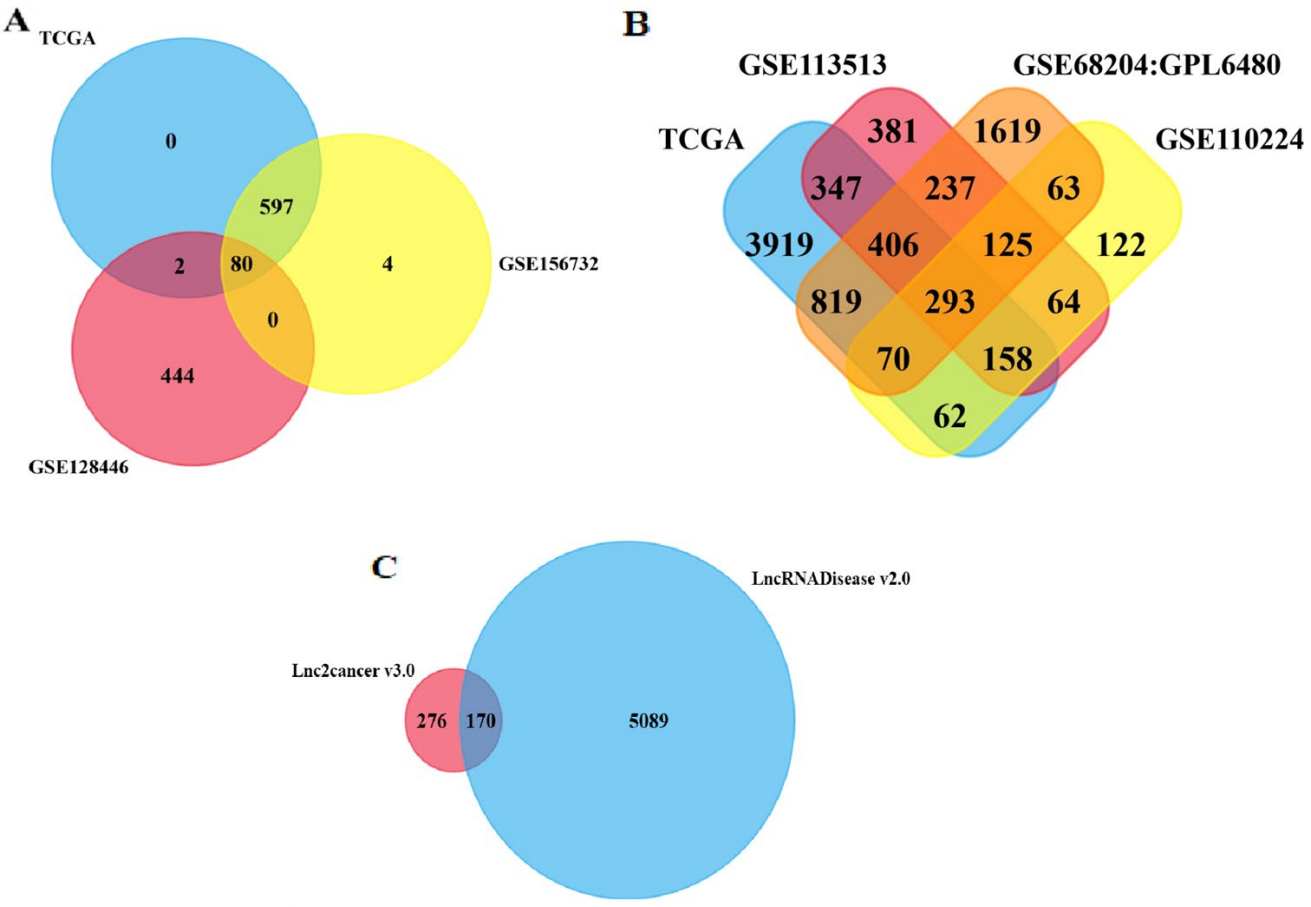
Venn diagram of the differently expressed miRs, lncRNAs, and mRNAs between GEO and TCGA datasets. Allocation of (**A**) the 80 differently expressed miRs (33 up-regulation and 47 down-regulation), (**B**) the 293 differently expressed genes (100 up-regulation and 193 down-regulation), and (**C**) the 170 differently expressed lncRNAs (111 up-regulation and 39 down-regulation) found between the selected datasets used in the present study.

GEO and TCGA datasets were analyzed to identify differentially expressed miRs, lncRNAs, and genes in colorectal cancer and normal samples. FunRich_3.1.3 software made a Venn diagram and extracted the common to the selected datasets (Fig. 1). A total of 80 miRs (33 up-regulation and 47 down-regulation) (Fig. 1A), 293 mRNAs (100 up-regulation and 193 down-regulation) (Fig. 1B), and 170 lncRNAs (111 up-regulation and 39 down-regulation) (Fig. 1C) were obtained from the selected datasets. The top up-regulated miRs were miR-21, miR-20a, and miR-20b, and the top down-regulated miRs were miR-34a, miR-424, and miR-378a (Table 1). The target genes of selected miRs have been represented in Tables 2, 3, and 4. Likewise, the lncRNAs of selected miRs were obtained using the LncRNADisease, Lnc2Cancer v3.0, LncRNA2target, and TANRIC datasets (Table 5).

**Table 1:** The predicted candidate miRs in rectal cancer patients.

miRs_ID	adj.P.Val
**Up-regulated**	
miR-21	2.16e−05
miR-20a	8.78e−04
miR-20b	1.39e−03
miR-424	1.93e−05
miR-1244	1.20e−03
miR-135b	4.20e−04
miR-224	3.60e−05
**Down-regulated**	
miR-378a	3.58e−04
miR-548ac	5.99e−03
miR-34a	2.97e−05
miR-133b	8.40e−06
miR-601	1.99e−05

**Table 2:** The predicted candidate genes in rectal cancer patients.

Up-regulated	AKT1, EGFR, IGF1, MET, TGFBR2, BCL2, ACVRIB, MAPK1, MAPK9, MYC, SMAD4, MAP2K1, FZD9, FZD4, FZD6, FZD10, MAP2KR1, KRAS, MAPK4, TGFBR2, IGF1R, PI3KR1, IGFB1, IGFB2, IGFBR2, APPL1, MAPK3, AKT2, AKT3, BRAF, GRB2, GRB10, CCND1, JUN, PDGFRA, DVL1, LEF1, BIRC5, CYCS
Down-regulated	APC, MSH2,MSH6, TP53, MSH3, TCF7L2, AXIN2, CTNNB1, PTEN, FOXO3, PDCD4, TCF7L2, E2F2, TCF7, BAK1, APC2, E2F1, GSK3B, BAX, SP1, RB1, GSK3B, SP1, AP1, TCF7, FRAT2, FRAT1, ESR1, ESR2, BAK1, DDB2, E2F3, ESRRG, ACVR1B, KIT, HEYL, GADD45A, CSNK1A1, CSNK1A1L, TNFSF11, SCH3, AXIN2, ZDHHC21

**Table 3:** Interaction analysis between the candidate miRs and target genes in rectal cancer patients.

miRs	Target genes
miR-21	BCL2, CCND1, CYCS, IGF1, MSH2, MYC, TGFBR2
miR-20a	AKT3, BCL2, CCND1, CYCS, MSH2, MYC, SMAD4, TGFBR2, TP53, IGF1, GRB10
miR-20b	CCND1, CYCS, MAPK4, MSH2, SMAD4, TGFBR2, IGF1, GRB10, AKT3
miR-378a	CYCS, IGF1, MYC
miR-424	AKT3, CCND1, GRB10, SMAD4
miR-1244	TGFBR2, GRB10
miR-34a	BCL2, CCND1, IGF1, MYC, SMAD4, TGFBR2, TP53, MSH2
miR-548ac	AKT3, MYC, BCL2, GRB10, IGF1, SMAD4,CCND1,CYCS, TGFBR2, MSH2, TP53
miR-135b	MYC, TGFBR2
miR-224	BCL2, GRB2, IGF1, SMAD4, CCND1, AKT3
miR-601	IGF1, SMAD4, TGFBR2, MSH2, GRB10
miR-133b	IGF1

**Table 4:** The candidate genes in rectal cancer patients.

Genes	adj.P-value
MAPK11	3.35e−33
AKT3	3.10e−40
MYC	9.16e−35
CCND1	8.53e−78
CYCS	1.60e−22
IGF1	2.78e−23
TGFBR2	4.68e−02
GRB10	1.92e−10
BCL2	7.19e−49
SMAD4	2.29e−02
TP53	2.46e−40
MSH2	2.38e−71

**Table 5:** The predicted candidate lncRNAs in rectal cancer patients.

LncRNAs	adj.P.Val
**Up-regulated**	
PVT1	0.00082474
HOTAIR	0.00070957
MALAT1	0.00095166
UCA1	0.00002353
CCAT1	0.00438593
CRNDE	0.0006234
XLOC-006844	0.0001641
LOC152578	0.0001971
XLOC-000303	0.00004521
BCAR4	0.0000532
**Down-regulated**	
LincRNA-P21	0.00027137

### Enrichment analysis of differentially expressed genes (DEGs)

**Figure 2:**
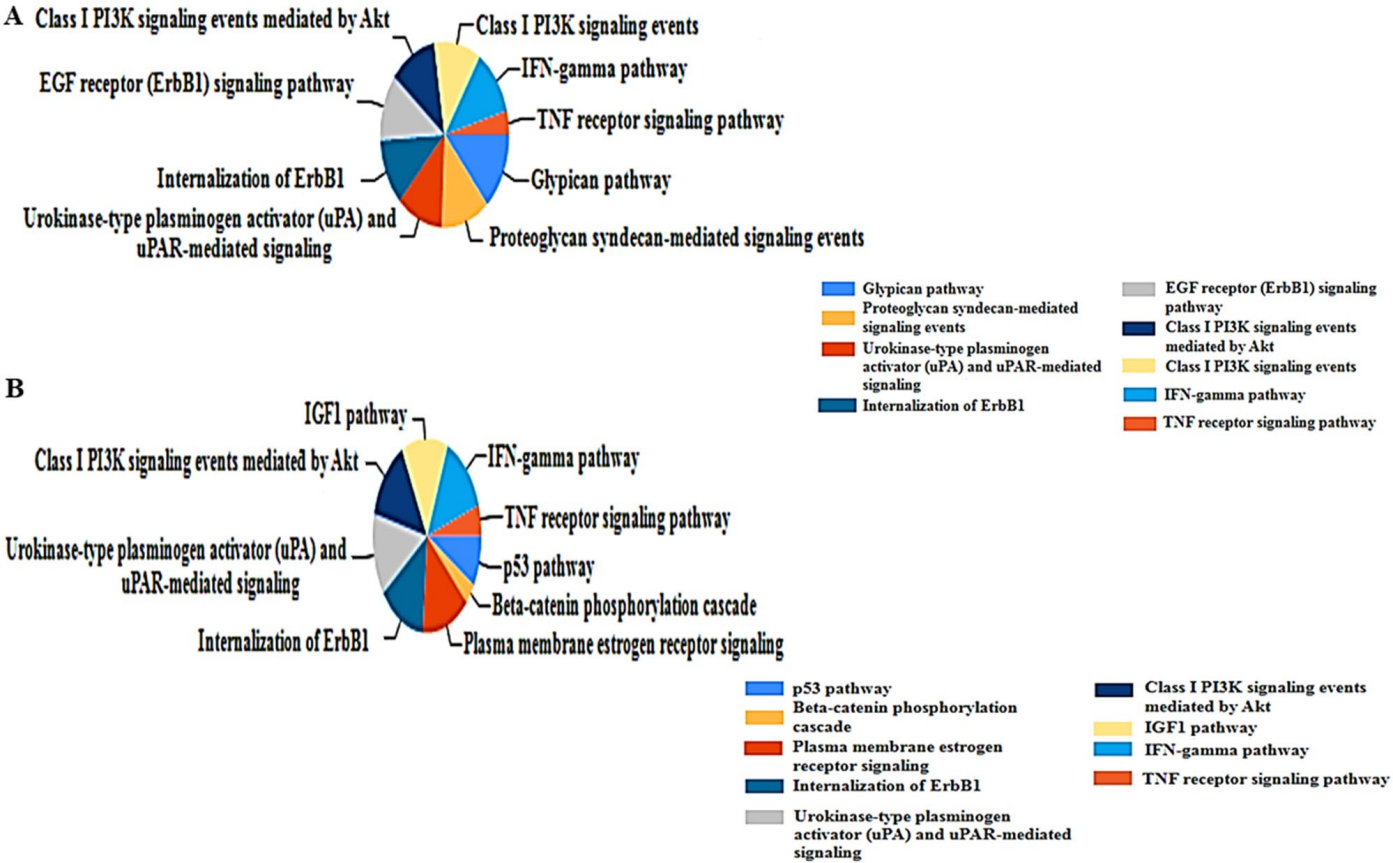
KEGG (Kyoto Encyclopedia of Genes and Genomes) pathway enrichment analysis of the DEGs. (**A**) Top 9 functional network/pathways associated with these up-regulated DEGs through KEGG analysis with a *p-*value of less than 0.05. (**B**) Top 9 functional network/pathways related to these down-regulated DEGs through KEGG analysis with a *p-value* of less than 0.05. Permission has been obtained from Kanehisa laboratories for using the KEGG pathway database^47^.

To examine the biological functions of the 293 DEGs, GO analysis was performed in the FunRich software. The up-regulated DEGs were enriched in the receptor binding, protein serine/threonine kinase activity, and growth factor activity (Table 6). In contrast, down-regulated DEGs' functional enrichment terms were mainly correlated with the transcription factor activity, kinase regulator activity, DNA binding, and DNA repair protein (Table 7). Up-regulated DEGs were enriched in the pathways, including the IFN-γ pathway, the Glypican pathway, and the TNF receptor signaling pathway (Fig. 2A). However, down-regulated DEGs pathways were enriched, including the IFN-γ pathway, IGF1 pathway, P53 pathway, and TNF receptor signaling pathway (Fig. 2B).

**Table 6:** GO (Gene Ontology) enrichment analysis for up-regulated DEGs.

Term name (Term ID)	adj.P	− log_10_(adj.P)
**GO: MF**		
Protein kinase activity (GO:0004672)	1.484 × 10^–11^	10.82853677
Phosphotransferase activity, alcohol group as acceptor (GO:0016773)	1.414 × 10^–10^	9.849644909
Kinase activity (GO:0016301)	9.313 × 10^–10^	9.030920019
Identical protein binding (GO:0042802)	1.614 × 10^–9^	8.792172425
Transferase activity, transferring phosphorus-containing groups (GO:0016772)	1.009 × 10^–8^	7.995924934
Protein serine/threonine kinase activity (GO:0004674)	2.402 × 10^–7^	6.619432031
Wnt-activated receptor activity (GO:0042813)	2.488 × 10^–6^	5.604201827
**GO: BP**		
Positive regulation of phosphorylation (GO:0042327)	1.628 × 10^–16^	15.78822386
Positive regulation of phosphate metabolic process (GO:0045937)	7.540 × 10^–16^	15.12264142
Positive regulation of phosphorus metabolic process (GO:0010562)	7.540 × 10^–16^	15.12264142
Gland development (GO:0048732)	1.261 × 10^–15^	14.89941364
Protein phosphorylation (GO:0006468)	2.501 × 10^–14^	13.60187147
Phosphorylation (GO:0016310)	4.032 × 10^–14^	13.39443361
Cell surface receptor signaling pathway (GO:0007166)	1.247 × 10^–13^	12.90412397
**GO: CC**		
Anchoring junction (GO:0070161)	5.902 × 10^–4^	3.229025174
Plasma membrane-bounded cell projection (GO:0120025)	1.881 × 10^–3^	2.725516421
Cell periphery (GO:0071944)	2.733 × 10^–3^	2.563400996
Cell projection (GO:0042995)	3.095 × 10^–3^	2.50936857
Plasma membrane (GO:0005886)	3.497 × 10^–3^	2.456283742
Early endosome (GO:0005769)	4.718 × 10^–3^	2.326204097
Cell junction (GO:0030054)	6.161 × 10^–3^	2.210340371

**Table 7:** GO (Gene Ontology) enrichment analysis for down-regulated DEGs.

Term name (Term ID)	adj.P	− log_10_(adj.P)
**GO: MF**		
Beta-catenin binding (GO:0008013)	1.768 × 10^–11^	10.75262
Enzyme binding (GO:0019899)	1.557 × 10^–7^	6.807835
Transcription factor binding (GO:0008134)	1.317 × 10^–6^	5.880288
Single guanine insertion binding (GO:0032142)	2.470 × 10^–6^	5.60732
Kinase binding ( GO:0019900)	2.608 × 10^–6^	5.583758
Double-stranded DNA binding (GO:0003690)	4.495 × 10^–6^	5.347278
Single base insertion or deletion binding (GO:0032138)	9.867 × 10^–6^	5.005819
**GO: BP**		
Canonical Wnt signaling pathway (GO:00600700	8.084 × 10^–11^	10.09239
Positive regulation of nitrogen compound metabolic process (GO:0051173)	4.183 × 10^–10^	9.37852
Wnt signaling pathway (GO:0016055)	8.050 × 10^–10^	9.094225
Cell–cell signaling by wnt (GO:0198738)	8.470 × 10^–10^	9.072135
Regulation of developmental process (GO:0050793)	1.049 × 10^–9^	8.979385
Beta-catenin destruction complex disassembly (GO:1904886)	3.510 × 10^–9^	8.454731
Apoptotic signaling pathway (GO:0097190)	4.886 × 10^–9^	8.311058
**GO: CC**		
Beta-catenin destruction complex (GO:0030877)	9.897 × 10^–12^	11.0045
RNA polymerase II transcription regulator complex (GO:0090575)	2.813 × 10^–7^	6.55087
Chromosome (GO:0005694)	7.278 × 10^–7^	6.138011
Chromatin (GO:0000785)	3.374 × 10^–6^	5.471906
Nuclear lumen (GO:0031981)	4.468 × 10^–6^	5.349878
Nucleoplasm (GO:000565)	6.181 × 10^–6^	5.208948
Transcription regulator complex (GO:0005667)	1.441 × 10^–5^	4.841472

### Protein–protein interaction (PPI) network analysis of DEGs

**Figure 3:**
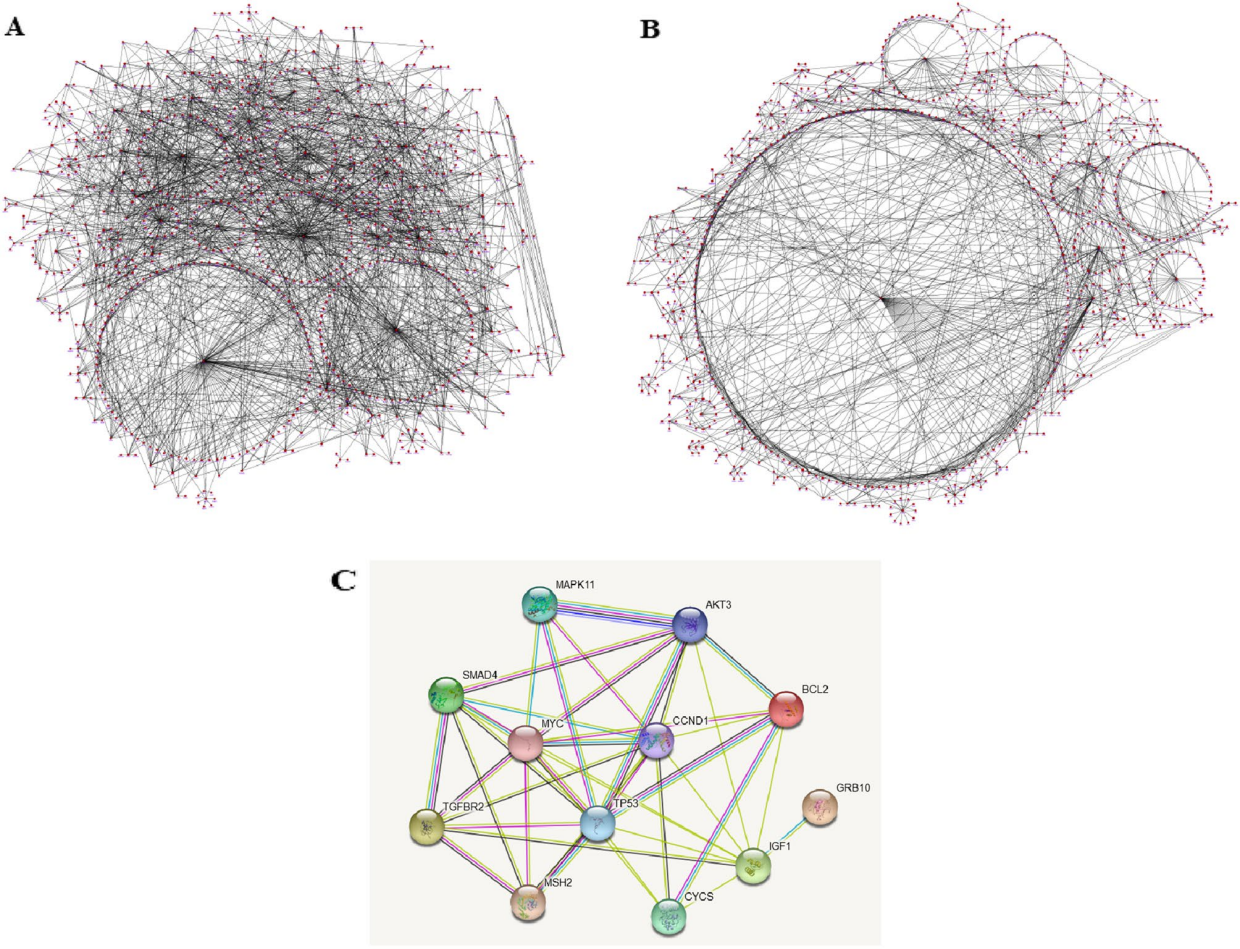
Protein–protein interaction (PPI) network construction. PPI network was constructed with the DEGs of GEO and TCGA datasets. (**A**,**B**) The significant module was identified from the PPI network using the FunRich software with a score of ≥ 7. Panel (**A**) shows the interaction between 193 down-regulated genes. Panel (**B**) shows the interaction between 100 up-regulated genes. Panel (**C**) shows the interaction between up-and down-regulated selected genes.

PPI analysis of the 293 DEGs was performed in the FunRich software (score ≥ 7). TP53, SP1, CTNNB1, ESR1, and GSK3B were hub nodes with higher node degrees in up-regulated genes (Fig. 3A). SMAD4, MAPK11, MYC, TGFBR2, GRB10, and MSH2 were hub node degrees in down-regulated genes (Fig. 3B). As a result, MAPK11, AKT3, MYC, CCND1, CYCs, IGF1, TGFBR2, GRB10, SMAD4, TP53, and MSH2 were selected as hub genes for further analysis owing to the high degree of connectivity (Fig. 3C).

### Construction of the lncRNA–miR–mRNA network

**Figure 4:**
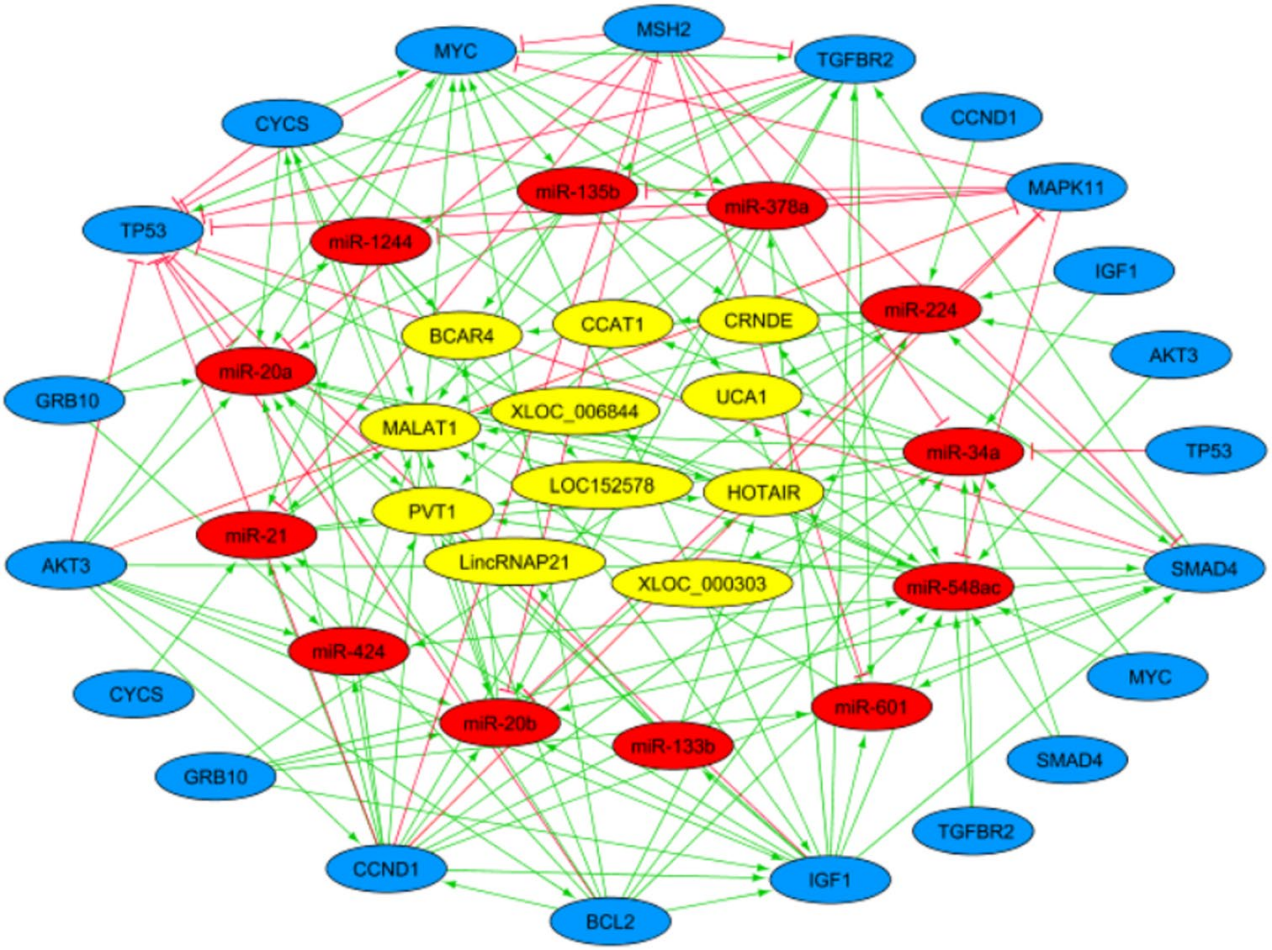
The lncRNA–miRNA–mRNA network. The network includes 44 nodes and 153 edges. The yellow, red, and blue ellipses represent the lncRNAs, miRs, and genes, respectively.

Figure 4 was created based on the lncRNA–miR–mRNA network that included 44 nodes and 153 edges. A total of 11 lncRNAs (Table 5), 12 miRs (Table 1), and 12 mRNAs (Table 4) was selected to construct the lncRNA–miR–mRNA network (Fig. 4).

### Experimental sampling

**Figure 5:**
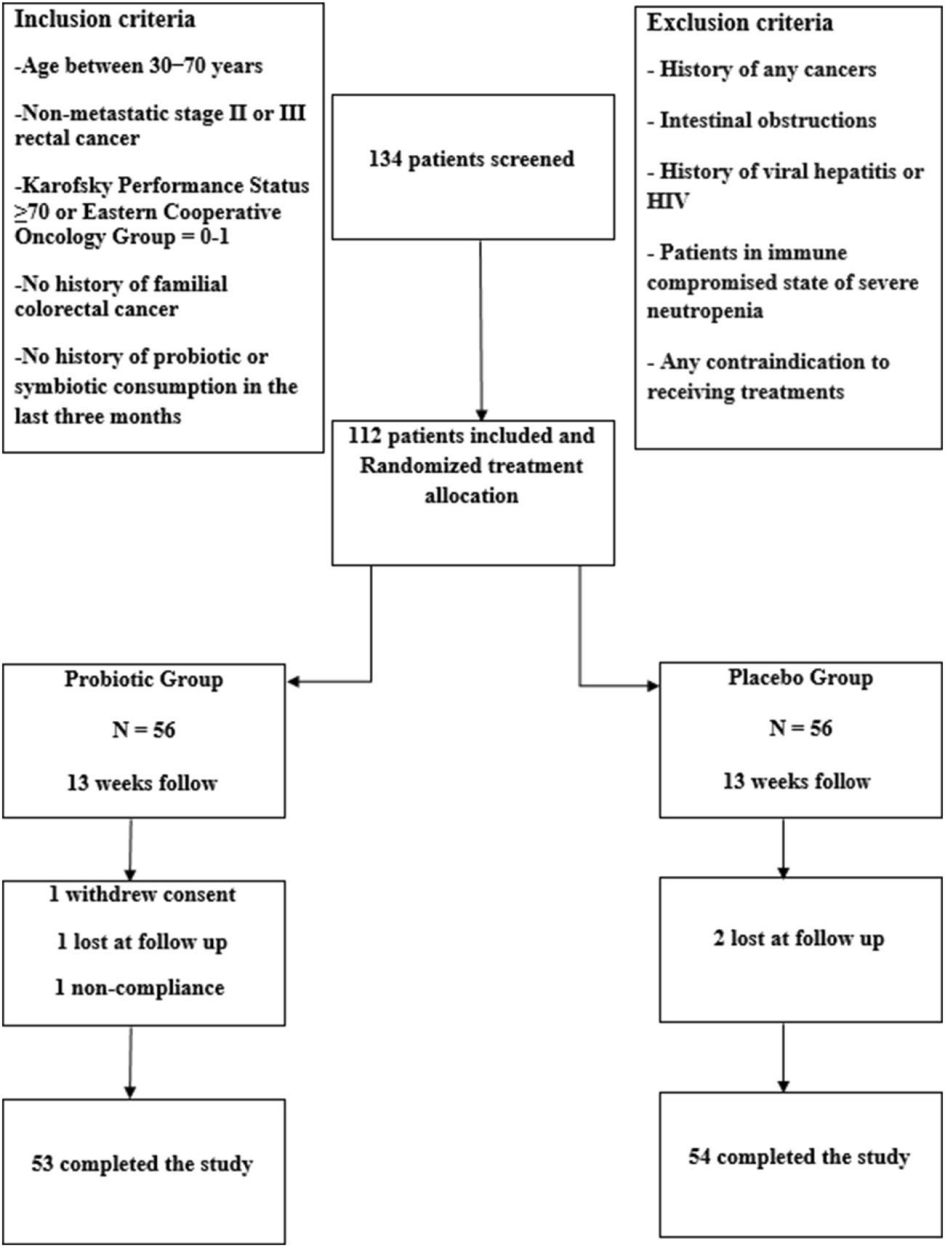
A flowchart of the present trial strategy.

The demographic characteristics of the participants have been summarized in Table 8. During the weeks of follow-up, 5 patients, consisting of 3 patients in the probiotic group and 2 patients in the placebo group, withdrew from the study (Fig. 5). Finally, 107 patients diagnosed with rectum cancer (probiotic group: 53, placebo group: 54) finished the examinations. The mean age was 57.3 ± 11.5 years, and the majority of the participants (59.7%) were male. Regarding the disease's staging, the diagnoses were made at stage III (70.9%) and stage II (29.1%).

**Table 8:** Baseline characteristics of the participants in the present study.

Variables	Probiotic	Placebo	Control	P-value
Sex (%)				0.9
Male	30 (56.6)	33 (61.1)	6 (60)	
Female	23 (43.4)	21 (38.8)	4 (40)	
Age (years) (mean ± SD)	51.3 ± 10.6	55.6 ± 10.5	52.3 ± 12.5	0.04
Height (cm) (mean ± SD)	165.6 ± 8.3	166.9 ± 8.0	168.7 ± 7.4	0.6
Weight (kg) (mean ± SD)	70.4 ± 10.1	72.8 ± 9.4	79.4 ± 10.4	0.3
BMI (kg/m^2^) (mean ± SD)	25.6 ± 3.9	26.1 ± 2.9	28.2 ± 4.4	0.5
Tumor stage (%)				0.9
2	11 (20.8)	10 (18.5)		
3	42 (79.2)	44 (81.5)		

*BMI* Body mass index.

### The expression of onco- and tumor suppressor lncRNAs in pre-and post-intervention

**Figure 6:**
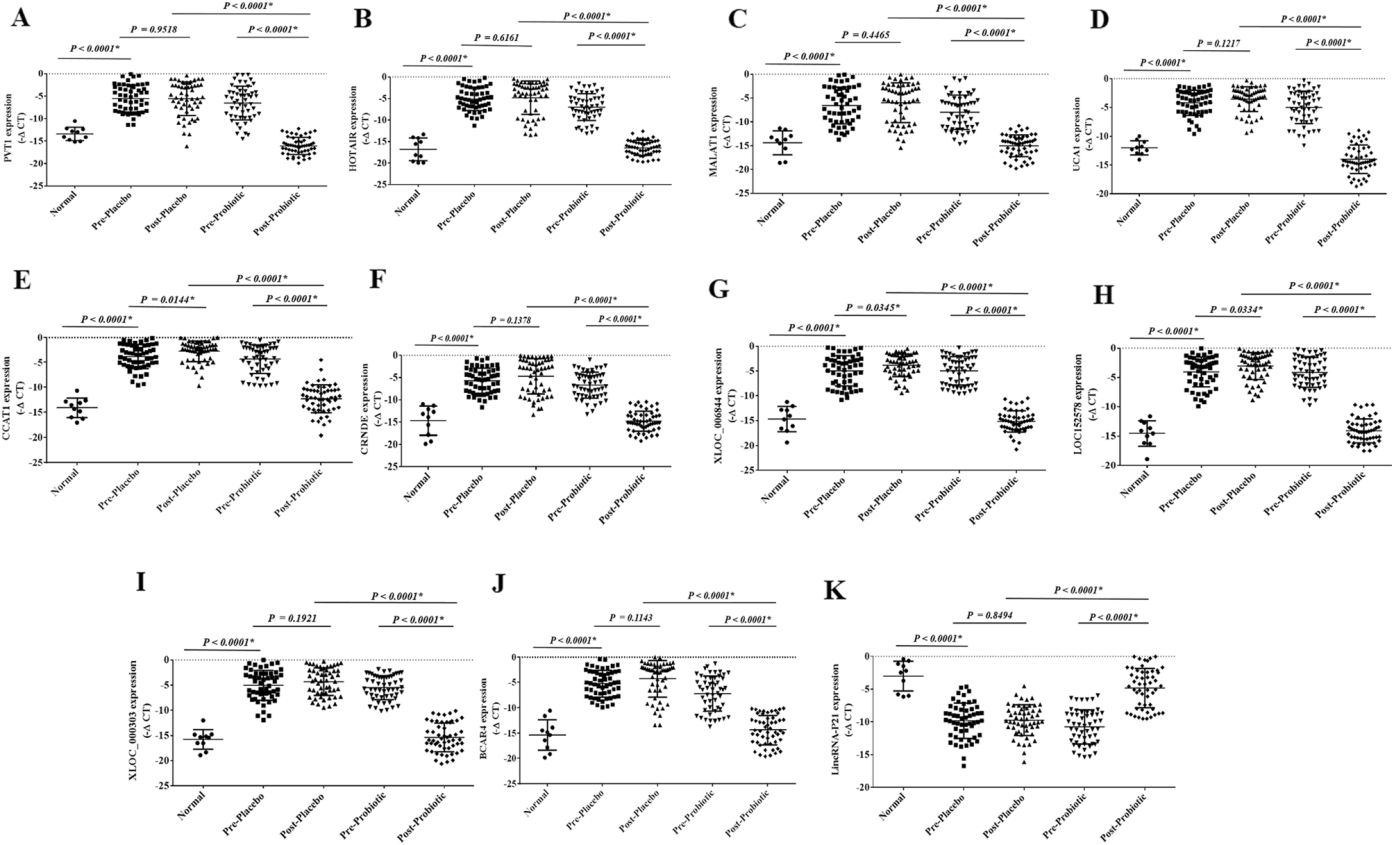
The relative expression of the selected lncRNAs in the rectal cancer patients. The relative expression levels of the lncRNAs were normalized by using a reference RNA. The oncogenic lncRNAs included: (**A**) PVT1, (**B**) HOTAIR, (**C**) MALAT1, (**D**) UCA1, (**E**) CCAT1, (**F**) CRNDE, (**G**) XLOC_006844, (**H**) LOC152578, (**I**) XLOC-000303, and (**J**) BCAR4. Tumor suppressor lncRNAs included: (**K**) LincRNA-P21.

The expression levels of the onco-lncRNAs, including CCAT1, LOC152578, UCA1, CRNDE, PVT1, MALAT1, XLOC_000303, XLOC_006844, BCAR4, and HOTAIR, were significantly increased in the rectal cancer patients compared to the control group. Their expression levels were significantly decreased following the probiotic consumption (Fig. 6A–J) (P < 0.05). Unlike CCAT1, LOC152578, and XLOC_006844, the expression levels of the other onco-lncRNAs did not exhibit significant changes after the placebo consumption. Nevertheless, the expression levels of the onco-lncRNAs were meaningfully lower in the probiotic users than the placebo group (P < 0.05).

The expression level of tumor suppressor lncRNA, including LincRNA-P21, was significantly decreased in rectal cancer patients compared to the control group. It was dramatically increased following the probiotic consumption and was considerably higher in the probiotic users compared to the placebo group (Fig. 6K) (P < 0.05).

### The expression of selected onco-and tumor suppressor miRs in pre-and post-intervention

**Figure 7:**
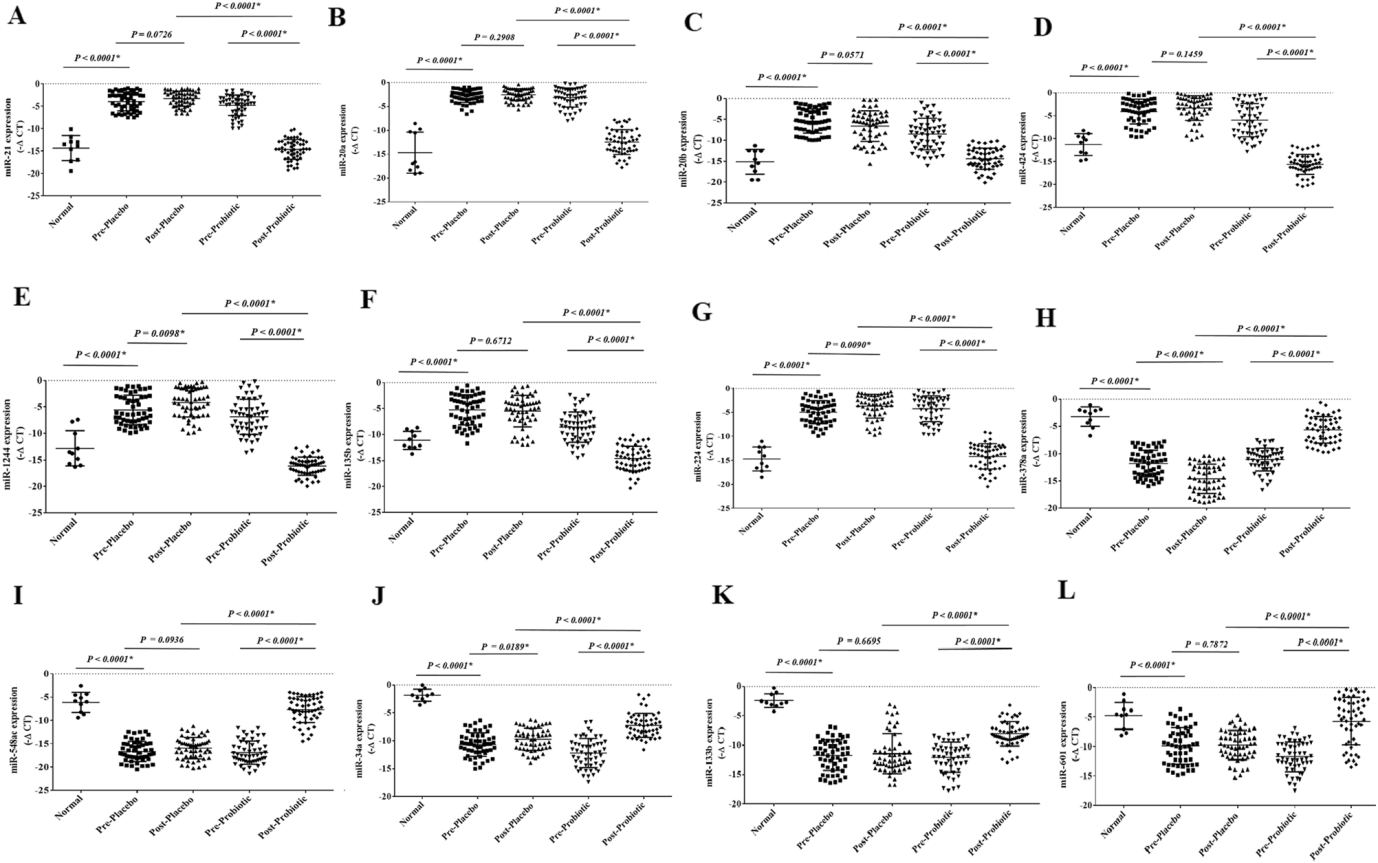
The relative expression of the selected miRs in the rectal cancer patients. The relative expression levels of the miRs were normalized by using a reference RNA. The oncomiRs included: (**A**) miR-21, (**B**) miR-20a, (**C**) miR-20b, (**D**) miR-424, (**E**) miR-1244, (**F**) miR-135b, and (**G**) miR-224. Tumor suppressor miRs included: (**H**) miR-378a, (**I**) miR-548ac, (**J**) miR-34a, (**K**) miR-133b, and (**L**) miR-601.

The expression levels of oncomiRs, including miR-21, miR-20a, miR-20b, miR-424, miR-1244, miR-135b, and miR-224, were significantly increased in the rectal cancer patients compared to the control group. Their expression levels were considerably decreased following the probiotic consumption (P < 0.05) (Fig. 7A–G). Unlike miR-1244, miR-378a, and miR-224, the other oncomiRs exhibited no significant changes after the placebo consumption. Notably, the expression levels of the oncomiRs were meaningfully lower in the probiotic group than the placebo (P < 0.05).

Our results showed that the expression levels of all selected tumor suppressor miRs, including miR-548ac, miR-378a, miR-34a, miR-601, and miR-133b, were significantly decreased in the rectal cancer patients compared to the control group, which were considerably increased following the probiotic consumption (P < 0.05) (Fig. 7H–L). Except for miR-378a and miR-34a, the levels of the other tumor suppressor miRs exhibited no significant changes after the placebo consumption. Notwithstanding, the expression levels of the tumor-suppressor miRs were meaningfully higher in the probiotic than the placebo users (P < 0.05).

### The expression of selected onco- and tumor suppressor genes in pre-and post-intervention

**Figure 8:**
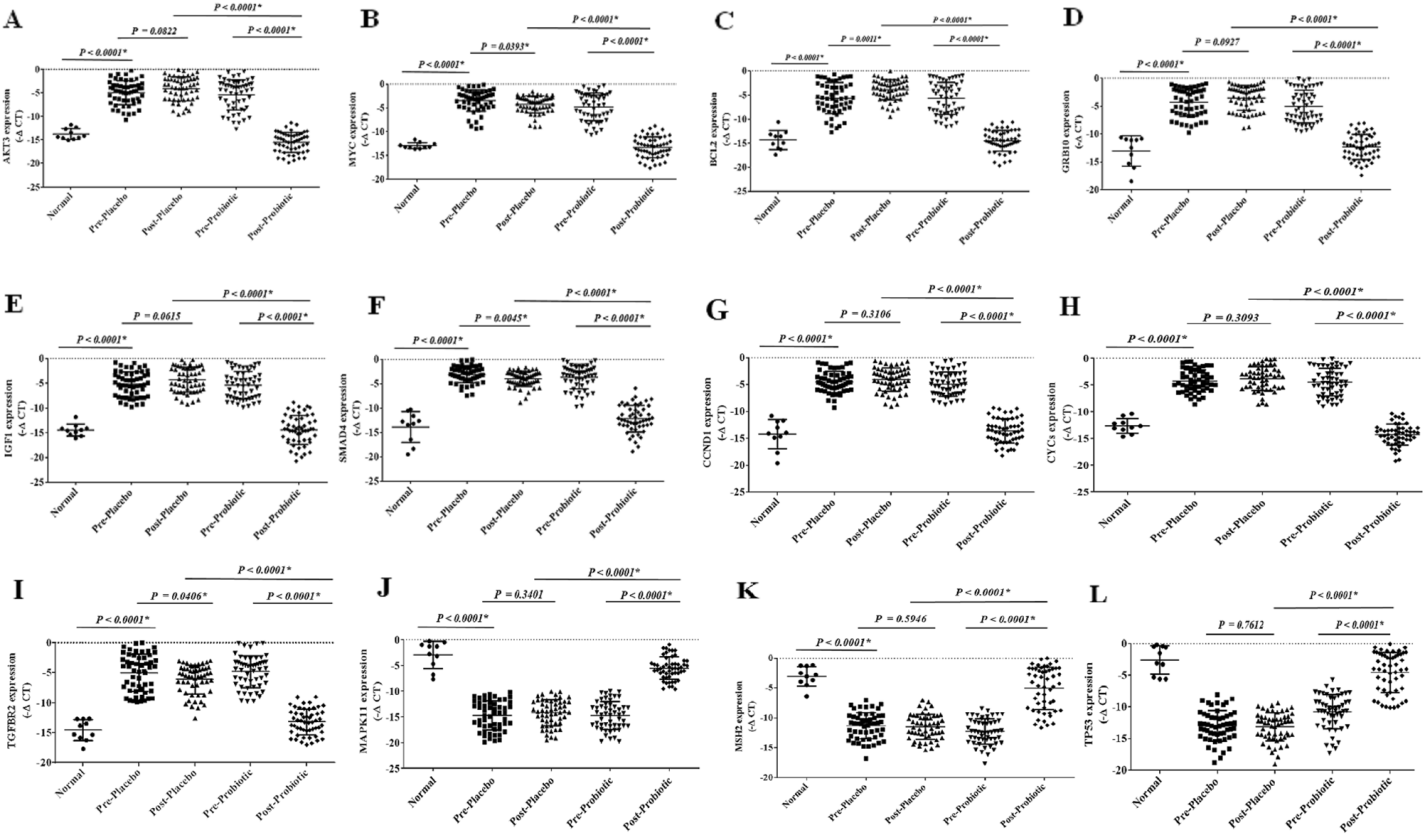
The relative expression of the candidate genes in rectal cancer patients. The relative expression level of the genes was normalized by using a reference gene. The oncogenes included: (**A**) AKT3, (**B**) MYC, (**C**) BCL2, (**D**) GRB10, (**E**) IGF1, (**F**) SMAD4, (**G**) CCND1, (**H**) CYCS, and (**I**) TGFBR2. Tumor suppressor genes included: (**J**) MAPK11, (**K**) MSH2, and (**L**) TP53.

The expression levels of oncogenes, including SMAD4, IGF1, GRB10, BCL2, CCND1, MYC, AKT3, TGFBR2, and CYCS, were significantly increased in the rectal cancer patients compared to the control group. Their expression levels were considerably decreased following the probiotic consumption (P < 0.05) (Fig. 8A–I). Except for BCL2, SMAD4, MYC, and TGFBR2, the other oncogenes revealed no significant changes after the placebo consumption (Fig. 8A–I). Nonetheless, the expression levels of the oncogenes were significantly lower in the probiotic group than the placebo (P < 0.05).

Moreover, our results showed that the tumor suppressor genes' expression levels, including MAPK11, TP53, and MSH2, were significantly decreased in the rectal cancer patients compared to the control group, which were considerably increased following the probiotic consumption (P < 0.05) (Fig. 8J–L). The placebo consumption did not significantly impact the selected tumor suppressor genes (Fig. 8J–L). Interestingly, the expression levels of the selected tumor suppressor genes were meaningfully higher in the probiotic group than the placebo group (P < 0.05).

### Correlation of the lncRNA–miR–mRNA network

**Figure 9:**
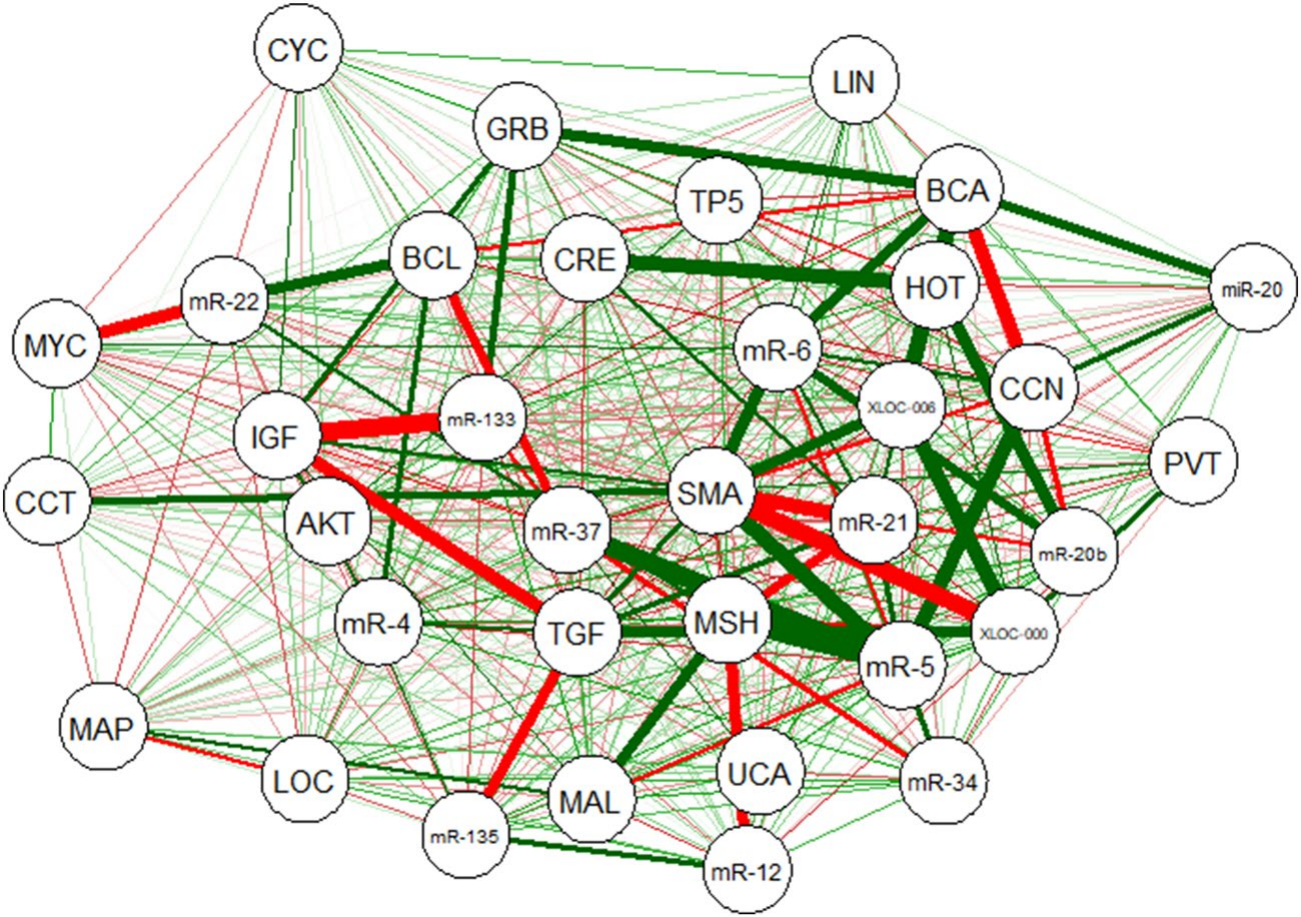
ceRNA regulatory network of lncRNAs, miRs, and mRNAs in rectal cancer. Red lines indicate a negative correlation, and green lines indicate a positive correlation. The figure was created using the R software: R Core Team (2019), R Foundation for Statistical Computing, Vienna, Austria. URL https://www.R-project.org/. PVT: PVT1, HOT: HOTAIR, MAL: MALAT1, UCA: UCA1, CCT: CCAT1, CRE: CRNDE, XLOC_006: XLOC_006844, LOC: LOC152578, XLOC-000: XLOC-000303, BCA: BCAR4, LIN: LincRNA-P21, MAP: MAPK11, AKT: AKT3, MYC: MYC, BCL: BCL2, GRB: GRB10, IGF: IGF1, SMA: SMAD4, CCN: CCND1, CYC: CYCS, TGF: TGFBR2, MSH: MSH2,TP5: TP53, mR-21: miR-21, mR-20: miR-20a, mR-20b: miR-20b, mR-4: miR-424, mR-12: miR-1244, mR-135: miR-135b, mR-22: miR-224, mR-37: miR-378a, mR-5: miR-548ac, mR-34: miR-34a, mR-133: miR-133b, mR-6: miR-601.

**Figure 10:**
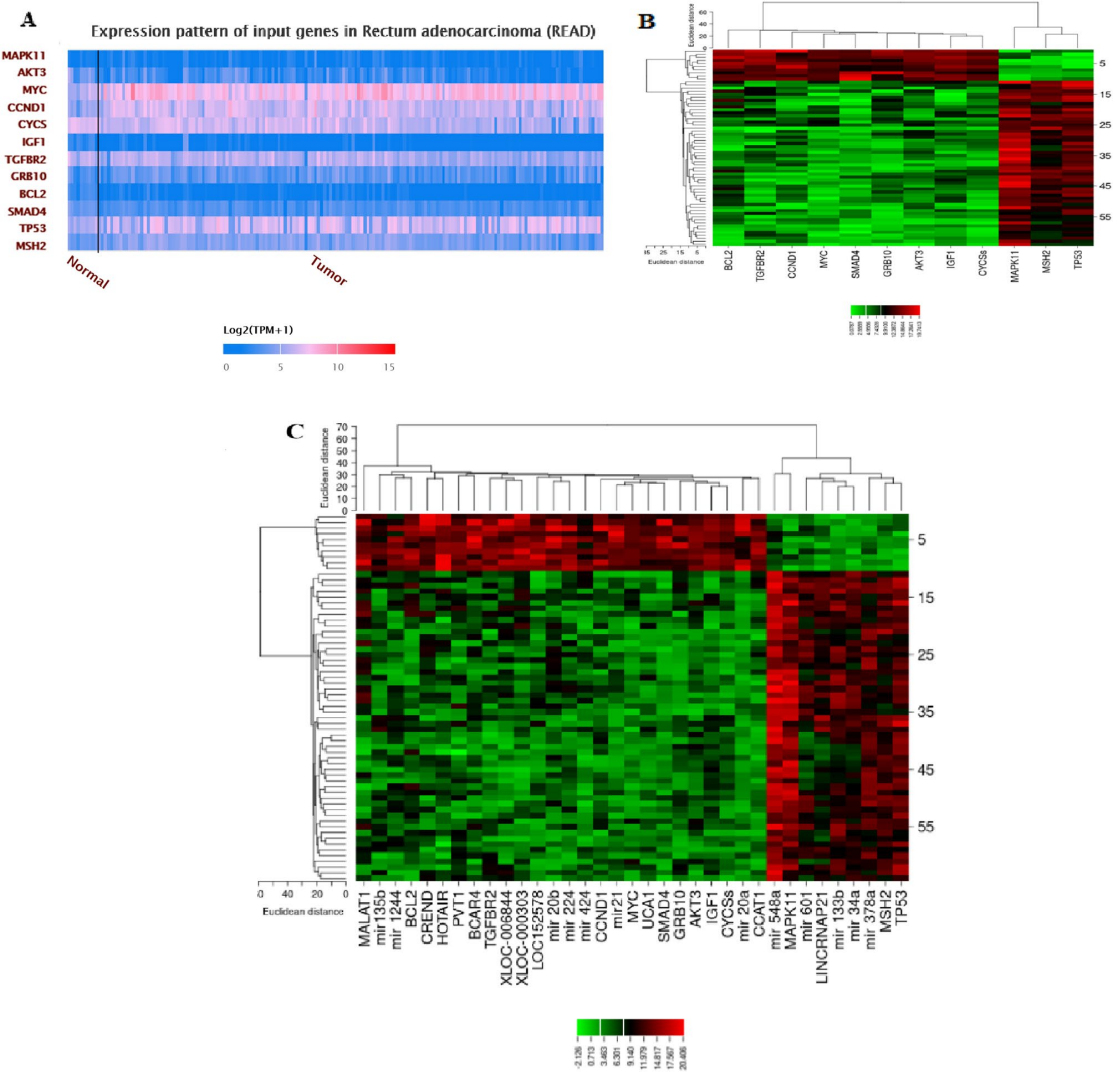
A plot heatmap to show the gene expression profile of DEGs in both bioinformatics (**A**) and experiment data (− ΔCT) (**B**,**C**).

To further understand the role of differential expression of ceRNAs in rectal cancer, we performed a correlation analysis between lncRNAs, miRs, and mRNAs. Consequently, 11 lncRNAs, 12 miRs, and 12 mRNAs constituted a direct regulatory relationship of the lncRNA–miRNA–mRNA network (Fig. 9). Accordingly, there was a strong correlation between some network components, including miR-133b and IGF1 gene, miR-548ac and MSH2 gene, and miR-21 and SMAD4 gene. Likewise, we created a heat map of the expression of the selected lncRNAs, miRs, and mRNAs using CIMminer (https://discover.nci.nih.gov/cimminer/home.do) (Fig. 10).

## Discussion

This study investigated the effects of *L.* *acidophilus* consumption on the expression of lncRNAs, miRs, and mRNAs in patients with non-metastatic rectal cancer. Based on a biphasic methodology, we constructed a network of the lncRNA–miR–mRNA using bioinformatics analyses. 11 lncRNAs, 12 miRs, and 12 genes have displayed significant differential expressions in cancerous tissues compared to noncancerous tissues. Besides, our experimental results have shown that the *L.* *acidophilus* consumption was associated with an expressional improvement of candidate lncRNAs, miRs, and mRNAs compared to the placebo group.

Some genes can play a role in tumorigenesis and the progression of colorectal cancer. Accordingly, TGF-βR2 is a trans-membrane serine-threonine kinase and is the only known receptor complex for TGF-β to be phosphorylated. It, in turn, may phosphorylate downstream proteins, including the SMAD, PI3K, p38MAPK, PKA, and RhoA, leading to inhibiting cell proliferation, inducing apoptosis, terminating differentiation, and maintaining genetic stability. Furthermore, CCND1 expression was significantly related to lymph nodes and distant metastases. There was a significant statistical correlation between the CCND1 gene and high stages in colorectal cancer^15^. Here, we observed that the probiotic consumers had a lower expression level of CCND1 than the placebo group. Similar to our results, tumor suppressor genes such as the MAPK can also be up-regulated in probiotic consumption^16^. Our analysis showed a considerable interaction between the candidate DEGs and miRs such as miR-21, miR-20a, and miR-34a. The miR-21 overexpression is associated with a non-complete response to preoperative chemo-radiotherapy in patients with rectal adenocarcinomas^17,18^. Moreover, it was reported that c-Myc up-regulates the miR-17 and down-regulates the angiogenesis inhibitors. Dews et al. showed that the overexpression of miR-20a is associated with reduced TGF-βR2 protein levels in colon cells. They represented that the TGF-βR2 can be a direct target of miR-17/20a. This inhibition would deactivate the downstream mediators such as SMAD and thrombospondin type I, which can be associated with inhibition of angiogenesis in tumor cells^19^. In this setting, the miR-34 family is a transcriptional target of the p53, directly suppressing a set of canonical Wnt genes and Snail, resulting in p53-mediated suppression of Wnt signaling and the EMT process. Kim et al. reported that p53 could regulate GSK-3β nuclear localization via miR-34-mediated suppression of Axin2 in CRC^20^.

Although there is a strong link between changes in the intestinal microbiome and rectal cancer, the potential mediators of these relationships are unclear. Accordingly, our bioinformatics study analyzed the lncRNA–miR–mRNA network of the essence in rectal carcinogenesis. In this setting, several lncRNAs can target one miR by inhibiting its expression through various mechanisms. According to our results, HOTAIR, as a lncRNA, can negatively regulate the expression of miR-203a-3p, miR-545, and miR-218, leading to EGFR and VOPP1 regulation, which can be found to be related to chemotherapy resistance in rectal cancer^21^. LncRNAs can bind to miR-34a and disrupt the regulation of miRs and target genes, including GAPLINC and SNHG7, which may increase, migrate, and invade rectal cancer cells^22,23^. While lncRNAs have the necessary pathological properties for appropriate biomarkers, their extraction and measurement are limited. As a result, although several studies reported differences in the expression of new lncRNA markers in human plasma and serum, others have difficulty replicating^21^. However, there is no doubt that lncRNAs and miRs are essential players in cancer pathology and can be a significant regulator of CRC's biology in cell cultures, animal models, and human samples. Future systematic and integrated analysis of different RNA molecules with potential cross-discussion may greatly help unravel the complex mechanisms of tumorgenesis and treatment of rectal cancer.

The experiments and trials regarding the beneficial effects of probiotics in the cancer region showed promising results. Commensal Lactobacillus species (such as *L.* *acidophilus*) are normal inhabitants of the natural microbiota^24^. Importantly, probiotics have been shown to reduce colon cancer incidence in animal models^10,25^. Oral administration of *L. acidophilus* has effectively reduced colon carcinoma growth, suggesting that its consumption was associated with suppressed tumor growth^10,26^. In an animal model, Chen et al. reported that *L.* *acidophilus* could induce apoptosis of colon cancer cells by down-regulating BCL-2 expression and up-regulating caspase-3 and -9^27^. Urbanska et al. explained that oral administration of *L.* *acidophilus* in a yogurt formulation in Apc (Min/þ) mice minimized intestinal inflammation and delayed overall polyp progression. They showed that pre-inoculation with *L.* *acidophilus* in Bulb/c mice resulted in retarding tumor volume growth, lowered histopathology scores, enhanced apoptosis of tumor cells, and down-regulated surface proteins' expression^25^. Besides, Yue et al. found that the metabolites of *L.* *acidophilus* could suppress the cell metastasis of colon cancer by inhibiting the VEGF/MMPs signaling pathway^28^. Agah et al. showed that *L.* *acidophilus* could induce a lower level of CEA and CA19-9 and a higher level of IFN-γ in the azoxymethane-induced colon cancer by altering the T-Cell signature to increase CD4+ and CD8+ cells^10,29^. Moreover, Caramés et al. revealed the anti-cancer effects of *L.* *acidophilus* by expressing antioxidant enzymes on an animal colon cancer^17^.

Accordingly, probiotics could alleviate the complication of CRC patients who underwent surgery or chemoradiation therapy^30^. Kim et al. found that radiation causes significant changes in the microbiome abundance and diversity^31^ that can influence the effectiveness of the anti-cancer treatments. Moreover, the immune microenvironment may modulate radiosensitivity related to radiation injury. Current evidence supports the use of probiotics as adjunctive therapy. They might have beneficial effects on some aspects of toxicity related to radiotherapy. It seems that probiotics could be safely administered even in neutropenia^32^. A meta-analysis study showed that probiotics could reduce the incidence of diarrhea induced by radiotherapy and have beneficial effects in preventing radiation-induced diarrhea, especially for grade ≥ 2 or 3 diarrhea. They may be a safe, promising therapeutic alternative for cancer patients suffering radiotherapy-induced diarrhea^33^. In addition, probiotics have been shown to reduce tumor recurrence rates and protect the intestinal mucosa's physical and biological barrier functions. They can improve the integrity of the intestinal epithelial layer and increase resistance to pathogenic colonization^34^. They can also produce a fasting-induced adipose factor, a gut radioprotector^35^. Moreover, two probiotic strains, including Lactobacillus *fermentum* and Lactobacillus *salivarius*, could re-establish miR-155 and miR-223 expression, preserve the mucosal barrier function, and relieve the DSS-induced colitis^36^. Tan et al. performed a comprehensive analysis of the lncRNA–miR–mRNA regulatory network for microbiota-mediated colorectal cancer^37^. They showed that probiotics could regulate lncRNAs' expression levels by competitively binding to the corresponding miRs and mRNAs, called ceRNA regulatory network^38^. These researchers identified a set of microbiota-mediated biomarkers and constructed ceRNA networks in CRC. Accordingly, 75 DELs, 8 DEMs, and 9 DEGs in the probiotic-related ceRNA network were obtained. They exhibited that the probiotics could inhibit the oncogenes' expression, including miR-153 and miR-429, and promote the tumor suppressors' expression, including miR-140 and miR-132^37^. They also showed that four lncRNAs from the microbiota-mediated ceRNA network, including LINC00355, KCNQ1OT1, LINC00491, and HOTAIR, were found to be associated with poor overall survival. These results could indicate a potential mechanism where probiotics can regulate immune system functions in CRC.

Here, we have demonstrated that the administration of probiotics could improve the molecular profile of rectal cancer patients. This novel effect yielded that probiotics could play more fundamental roles in CRC management and co-administration with chemoradiation therapies to reduce complications and increase their efficacy. Likewise, similar outcomes were pursued by an unpublished study (NCT03072641) aiming to determine if probiotics could alleviate the cancer-associated gut microbiota and epigenetic alterations in CRC. Moreover, Zaharudinn et al. reported that the probiotics containing six viable microorganisms could reduce the post-surgical pro-inflammatory cytokines such as TNF-α, IL-6, IL-10, and IL-12 in CRC patients^39^. However, comprehensive research should be assumed better to understand the clinical values of probiotics in colorectal cancer. Therefore, it will be with much more clinical efficacy if the clinicians and researchers apply mechanism-oriented and population-specific approaches when dealing with probiotics.

## Conclusion

During radiotherapy, *L.* *acidophilus* consumption in rectal cancer patients for 13 weeks could reduce oncogenic lncRNAs, miRs, and mRNAs and simultaneously increase tumor-suppressor lncRNAs, miRs, and mRNAs. Our results suggest interactions among lncRNAs, miRs, and genes may mediate host-microbial interactions in rectal cancer and can be an explicit goal for developing treatment strategies. Moreover, promising therapeutic approaches for activating endogenous miR expression to mediate lncRNA silencing mediated by target miRs have been proposed, although more works need to be evaluated.

## Materials and methods

### Study setting

This study is part of an ongoing randomized clinical trial registered in the Iranian randomized control trial (NO: IRCT2014092118745N3). The study is a randomized, double-blind, and single-center conducted on 107 new cases (55 males and 52 females) with non-metastatic rectal cancer at Emam-Khomeini Hospital, Tehran, Iran, which entered into the study on 08-11-2014 (Fig. 5). All participants were informed of the current research objectives, study protocol, and informed consent to participate in the study.

### Study population

After reviewing the medical records of patients who had previously been confirmed diagnosing rectal cancer based on pathologic reports, the eligible cases were recruited. Inclusion criteria comprised age between 30 and 70 years, non-metastatic stage II or III rectal cancer, Karofsky Performance Status ≥ 70 or Eastern Cooperative Oncology Group = 0–1, no history of familial colorectal cancer, and no history of probiotic or symbiotic consumption in the last three months. Exclusion criteria included the history of other cancers, intestinal obstructions, viral hepatitis or HIV history, and patients with severe neutropenia.

### Randomization, allocation, and interventions

Randomization was performed on block randomization. The block randomization was performed based on blocks of 2, and the computer program performed it. Sealed envelopes with the treatment codes were stored in the same department. The patients were blinded using an identical capsule to those given to the intervention group as a placebo. Besides, the caregiver and the laboratory staff were all blinded to the patient’s medical documents. Similarly, the statistician who performed the statistical analyses was also blinded to the grouping codes assigned in the dataset. The patients in the probiotic group received probiotic capsules (500 mg) (10^9^ CFU) for 13 weeks, taking the tablets three times a day. The subjects in the placebo group received placebo capsules with the same shape, color, and smell as the probiotic group's protocol.

### Primary outcomes

We measured and compared the expression levels of candidate lncRNAs (Table 5), miRs (Table 1), and mRNAs (Table 4) before (baseline) and after three months of the intervention in the probiotic and placebo groups.

### Identification of differentially expressed genes, miRs, and lncRNAs in rectal cancer datasets

**Figure 11:**
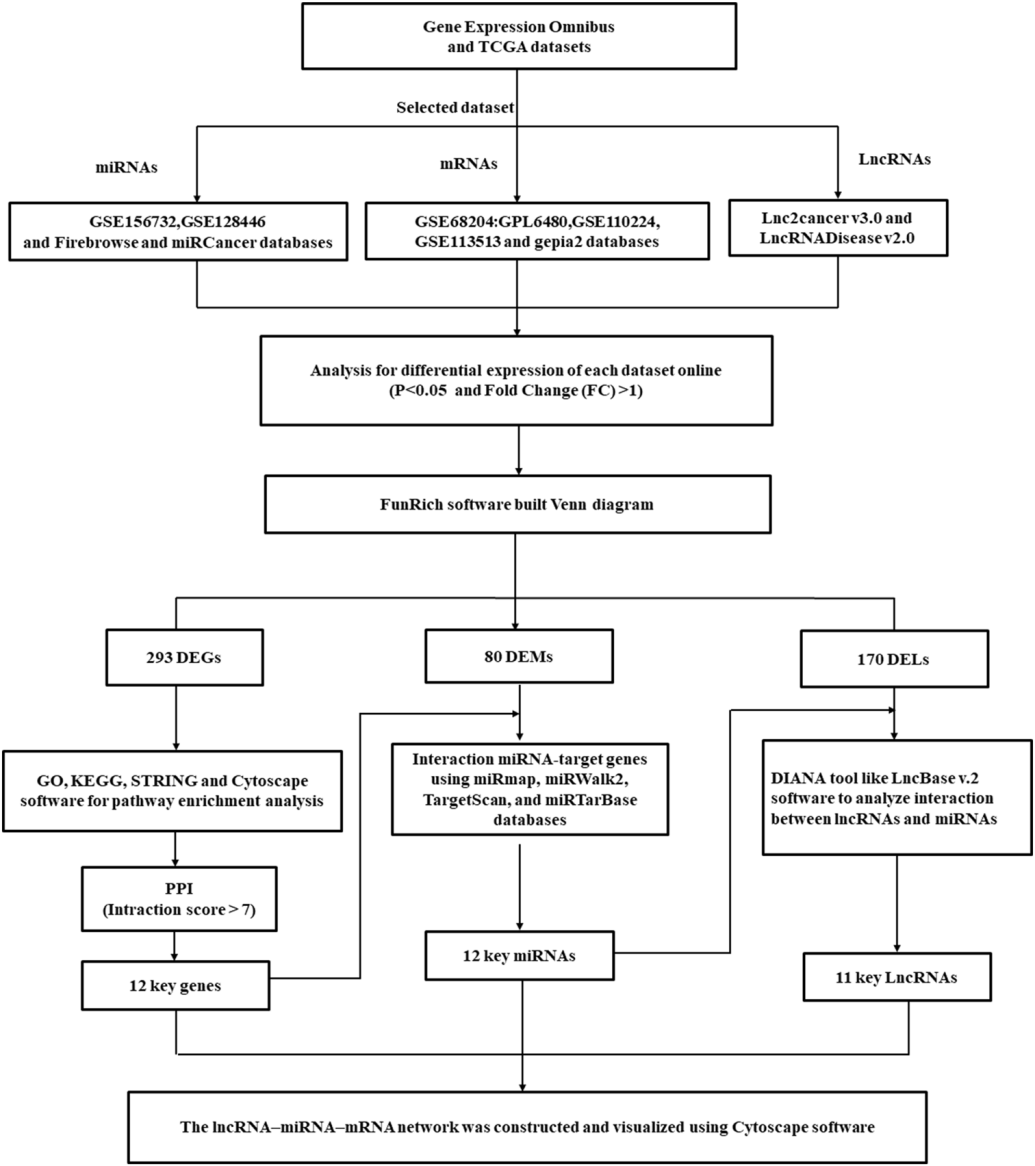
A flowchart diagram for used bioinformatics analysis in the present study.

The platforms used for miRs and mRNAs, including the miRs' expression profile (GSE128446 and GSE156732) and the mRNAs' expression profile (GSE68204: GPL6480, GSE110224, and GSE113513), were downloaded from the Gene Expression Omnibus (GEO) database (https://www.ncbi.nlm.nih.gov/geo). GSEs' data were downloaded for use with the GEOquery R package (https://bioconductor.org/packages/GEOquery)^40^. We analyzed the candidate miRs, lncRNAs, and genes with P-value < 0.05 and |LogFC|> 1 in the dataset as DEGs, differentially expressed miRNAs (DEMs), and differentially expressed lncRNAs (DELs). Moreover, the GEPIA2 (http://gepia2.cancer-pku.cn), the cBioPortal (https://www.cbioportal.org), and the Broad Institute’s FireBrowse (http://firebrowse.org) are websites for analyzing the differential expression genes from the TCGA and Genotype-Tissue Expression projects^41^. The used platforms for miRs included the OncomiR (http://www.oncomir.umn.edu/omcd/), miRGator 3.0 (https://tools4mirs.org), and miRCancerdb (http://mircancer.ecu.edu) databases of TCGA dataset^42^. The used databases for lncRNAs included LncRNADisease (http://www.rnanut.net/lncrnadisease) and Lnc2Cancer v3.0 (http://bio-bigdata.hrbmu.edu.cn/lnc2cancer) of TCGA dataset^43,44^. Figure 11 shows a flowchart diagram for used bioinformatics analysis.

### Predicted target genes and lncRNAs of candidate miRs

The target genes of miRs were identified using online predictive programs such as miRmap (https://mirmap.ezlab.org/app/), miRWalk2 (http://zmf.umm.uni-heidelberg.de/apps/zmf /mirwalk2/), TargetScan Release 7.0 (http://www.targetscan.org), and miRTarBase (https://mirtarbase.cuhk.edu.cn/miRTarBase) datasets. Furthermore, the lncRNAs that regulate DEMs were collected by the LncRNA2target, TANRIC, and lncBase (https://diana.e-ce.uth.gr/lncbasev3) datasets. The lncRNA–miR–mRNA network was constructed by the selected miRs, lncRNAs, and mRNAs. The results were visualized by Cytoscape_v3.1 (https://cytoscape.org) software^45^.

### Construction and analysis of the lncRNA–miR–mRNA network

The lncRNA–miR–mRNA network was constructed and visualized using Cytoscape software based on the ceRNA theory. Here, the nodes and edges were used to represent extensive biological data. Intuitively, each node represents a biological molecule, and the edges stand for the interactions between nodes and the node degrees. The edges were calculated to exploit the hub nodes that possess essential biological functions^46^. A network analysis was performed using Cytoscape software to explore the structure and feature of the lncRNA–miR–mRNA competing triplets. Topological parameters of standard centrality measures in a network, including DC, BC, and CC, were assessed. The DC is defined as the number of links incident upon a node. The BC for each node is calculated as the number of these shortest paths that pass through the node. The CC is the length of the shortest paths between the node and other nodes in the network.

### Correlation of lncRNA–miR–mRNA network

We constructed a heat map based on our experimental data to show the possible correlation of the selected lncRNAs, miRs, and mRNAs. The absolute value of the correlation coefficient (equal to or more than 0.5) represented a significant correlation. We matched interactions of miRs according to the miR-code database (http://www.mircode.org/) by the differentially expressed lncRNA and miRs. Moreover, the target genes of miRs were created using the mentioned databases. At last, a ceRNA network between lncRNAs, miRs, and mRNAs was constructed using Cytoscape 3.1 software.

### The analysis of the GO term and KEGG pathways by FunRich software

The FunRich (http://www.funrich.org) is software for functional gene classification. The GO and KEGG (map05210)^47^ enrichment analyses of the DEGs were executed through the FunRich software.

### Sample collection

Before and after the intervention, 10 ml blood samples were obtained from all the subjects using disposable vacutainer blood collection tubes. The blood was centrifuged at 3000*g* for 5 min, and peripheral blood mononuclear cells (PBMCs) were then isolated by the Ficoll-Hypaque (Amersham). The cells were suspended into 90% Foetal Bovin Serum (FBS) (life tech)/10% Dimethyl sulfoxide (DMSO) (Sigma), and the plasma and PBMCs were then preserved at − 80 °C.

### Real-time PCR analysis

According to the manufacturer's instructions, the total RNA was extracted from the plasma and PBMC samples. Plasma (250 μl) and PBMC (500 μl) were added to 750 μl and 500 μl TRIzol (Beijing Tiangen Biotech Co., Ltd.), respectively. The absorbance ratio (A260/280) of total RNA, between 1.8 and 2.2, was determined using an ultraviolet (UV) spectrophotometer. According to the manufacturer's instructions, the miRcute miRNA cDNA First-Strand Synthesis kit (Beijing Tiangen Biotech Co., Ltd) to quantify miRs and the cDNA Synthesis Kit (TAKARA BIO INC. Cat. 6 30 v.0708) to quantify genes and lncRNAs were used. Then, cDNA was used in each Real-Time PCR assay with the miRcute miRNA Fluorescence Quantitative Detection kit (Tiangen Biotech Co., Ltd.). The cycling conditions were pre-denaturation at 94 °C for 2 min, followed by 40 cycles of 94 °C for 20 s and 60 °C for 34 s. The SYBR Green method (AccuPower Green Star qPCR Master Mix; Bioneer, Korea) was used for genes and lncRNAs.

PCR cycling was performed for one cycle at 95 °C for 10 min, 40 cycles at 95 °C for 20 s, and 60 °C for 45 s. The melting curve analysis was run from 60 to 95 °C to confirm specific amplification^48,49^. The expression of U6 and B-actin was used to normalize miRs, lncRNAs, and genes as the Internal Reference Gene. The list of primers has shown in Table 9. The qRT-PCR reactions were performed using an ABI StepOne plus System (Applied Biosystems; Thermo Fisher Scientific, Inc). The expression level of the genes was calculated using the − ΔCT method. ΔCT was calculated by subtracting the CT values of U6 and B-actin from the CT values of the targets^50^. The expression data generated from our study samples have been available as a supplementary information file (Supplementary Information).

**Table 9:** The used primers for real-time PCR.

Genes/miRs/lncRNAs	Forward	Reverse
MAPK11	CTGAACAACATCGTCAAGTGCC	CATAGCCGGTCATCTCCTCG
AKT3	TGAAGTGGCACACACTCTAACT	CCGCTCTCTCGACAAATGGA
SMAD4	ACGAACGAGTTGTATCACCTGG	TGCACGATTACTTGGTGGATG
CCND1	CAATGACCCCGCACGATTTC	CATGGAGGGCGGATTGGAA
CYCS	CTTTGGGCGGAAGACAGGTC	TTATTGGCGGCTGTGTAAGAG
IGF1	TCGACATCCGCAACGACTATC	CCAGGGCGTAGTTGTAGAAGAG
TGFBR2	GCTTTGCTGAGGTCTATAAGGC	GGTACTCCTGTAGGTTGCCCT
BCL2	TCGCCCTGTGGATGACTGA	CAGAGACAGCCAGGAGAAATCA
GRB10	CTCGTGGCAATGGATTTTTCTG	TCACTGTACTTAGGGTAGAAGGG
MYC	CACACCCACAATTCAGGAAGAG	GACGTGCTACAAGGTGGCA
TP53	ACTTGTCGCTCTTGAAGCTAC	GATGCGGAGAATCTTTGGAACA
MSH2	GATCAATCCCCAGTCTGTTGTT	CCAAAATCCACACTTGGCAAAA
B-actin	CACCATTGGCAATGAGCGGTTC	AGGTCTTTGCGGATGTCCACGT
miR-21	CCTTTAGGAGCATTATGAGC	CCATAAAATCCTCCCTCCA
miR-20b	ACACTGCACAGTCCCCACCATCT	GCCCTAAATGCCCCTTCTGGCA
miR-20a	ACACAGCTGGATGCAAACCTGCAA	AACTCCAGCTTCGGCCTGTCG
miR-224	AAAAGTAATTGCGAGTTTACC	ACAGCACCGCCTGGATAG
miR-548ac	CAGCTGGGTGCTCAGCCAG	GGCAACTTAATGTTTCTTGC
miR-135b	GTAGATCAGGGTCAGGAAC	CTCGTAGGTGCAAACACCAT
miR-133b	TGGTCAAACGGAACCAAGTC	TTGCCAGCCCTGCTGTAG
miR-378a	GGCCCAACTTGGGAAATGTA	GCAGGAACAACCAGAACATC
miR-424	GCAGCTCCTGGAAATCAA	CTCTCCTCGACTCGCAC
miR-34a	TGAGGGCGGCTGGGAAAGTG	TTCTCCCAGCCAAAAGCCGCC
miR-1244	AAGTAGTTGGTTTGTATGAG	GTCGTATCCAGTGCAGGGTCCGAGGT
miR-601	CAGCAAGGCGGCATCTC	GTGCGTGTCGTGGAGTCG
HOTAIR	GAGTTCCACAGACCAACACC	AATCCGTTCCATTCCACTGC
CCAT1	GGAGCATTCACTGACAACATC	TTAGCCATACAGAGCCAACC
UCA1	CGGCTTAGTGGCTGAAGAC	ATTGAGGCTGTAGAGTTTGAGG
PVT1	CTGGACGGACTTGAGAACTG	CAGCAACAGGAGAAGCAAAC
CRNDE	TTCTCTTGTAGGATGCCACTG	TTCTGCGTGACAACTGAGG
MALAT1	GCTCAGTTGCGTAATGGAAAG	GCTGCCTCAATGCCTACC
BCAR4	GCTGGAATACAATGGCGTAATC	TCAGAGCAAGACAAGCATCG
XLOC_006844	AGGGAAAAGTCAATGCCAGT	GATCTCAGGCACATACACAGC
LOC152578	GGAGAACGAAGGTGGTAACAG	GGGAGAAGCAGGATTTAGGATG
XLOC_000303	CCCTGTTGATTGACTTGTCTTG	CTTCTCTTGCTGTCTCCTACC
LinRNAP21	TCTTGTGGTGGTAAAGACA	CCTCAATGCAGGCATACACAT
U6	ATGCAGTCGAGTTTCCCACAT	CCATGATCACGAAGGTGGTTT

### Statistical analysis

The statistical analysis was carried out using GraphPad Prism 6.01 (https://www.graphpad.com) software. The one-sample K–S test was used to evaluate the normality of the data. The t-test and ANOVA were used to analyze the data in two and multiple groups. The descriptive analysis for quantitative data was performed using mean ± SD. The same analysis was performed for qualitative data by representing the frequencies and regarded percentages. We constructed a correlation network of the selected lncRNA–miR–mRNA using the R Core Team (2019), R Foundation for Statistical Computing, Vienna, Austria. URL https://www.R-project.org. The statistical significance was defined as P < 0.05.

### Ethical approval

All methods were performed under the relevant guidelines and regulations. All procedures performed in studies involving human participants were under the ethical standards of the institutional and/or national research committee and with the 1964 Helsinki declaration and its later amendments or comparable ethical standards. The experimental procedures and care protocols were approved by a review board committee of Tehran University of Medical Sciences and Alborz University of Medical Sciences (NO: IR.TUMS.IKHC.REC.1397.036 and IR.ABZUMS.REC.1398.154) and registered by the Iranian Randomized Control Trial (IRCT) ethical board (NO: IRCT2014092118745N3). Written informed consent was obtained from each participant before the sample collection.

## Supplementary Information

Supplementary Information.

## Data Availability

The data that support the findings of this study are available from the corresponding author, AMA, upon reasonable request.
